# Supplementary material for: Structure-Based High-Throughput Virtual Screening and Molecular Dynamics Simulation for the Discovery of Novel SARS-CoV-2 NSP3 Mac1 Domain Inhibitors
Source: Viruses. 2023 Nov 22;15(12):2291. doi: 10.3390/v15122291 (PMC10747130; doi:10.3390/v15122291)
Supplement: Supplementary file 1 [file viruses-15-02291-s001.zip › viruses-2695072-supplementary11.pdf]

viruses

MDPI

Supplementary Materials

Structure-Based High-Throughput Virtual Screening and Molecular Dynamics Simulation for the Discovery of Novel SARS-CoV-2 NSP3 Mac1 Domain Inhibitors

Behnaz Yazdani [1], Hajar Sirous [2,*], Simone Brogi [2,3,*] and Vincenzo Calderone [3]

[1] Bioscience Department, Faculty of Science and Technology (FCT), Universitat de Vic - Universitat Central de Catalunya (Uvic-UCC), 08500 Vic, Spain; behnazyazdani6@gmail.com (B.Y.)
[2] Bioinformatics Research Center, School of Pharmacy and Pharmaceutical Sciences, Isfahan University of Medical Sciences, Isfahan 81746-73461, Iran;
[3] Department of Pharmacy, University of Pisa, Via Bonanno 6, 56126, Pisa, Italy; vincenzo.calderone@unipi.it (V.C.)
* Correspondence: h_sirous@pharm.mui.ac.ir (H.S.); simone.brogi@unipi.it (S.B.)

Table of Contents

Figure S1 S2

Figure S2 S3

Figure S3 S4

Figure S4 S5

Figure S5 S6

Figure S6 S7

Figure S7 S8

Figure S8 S9

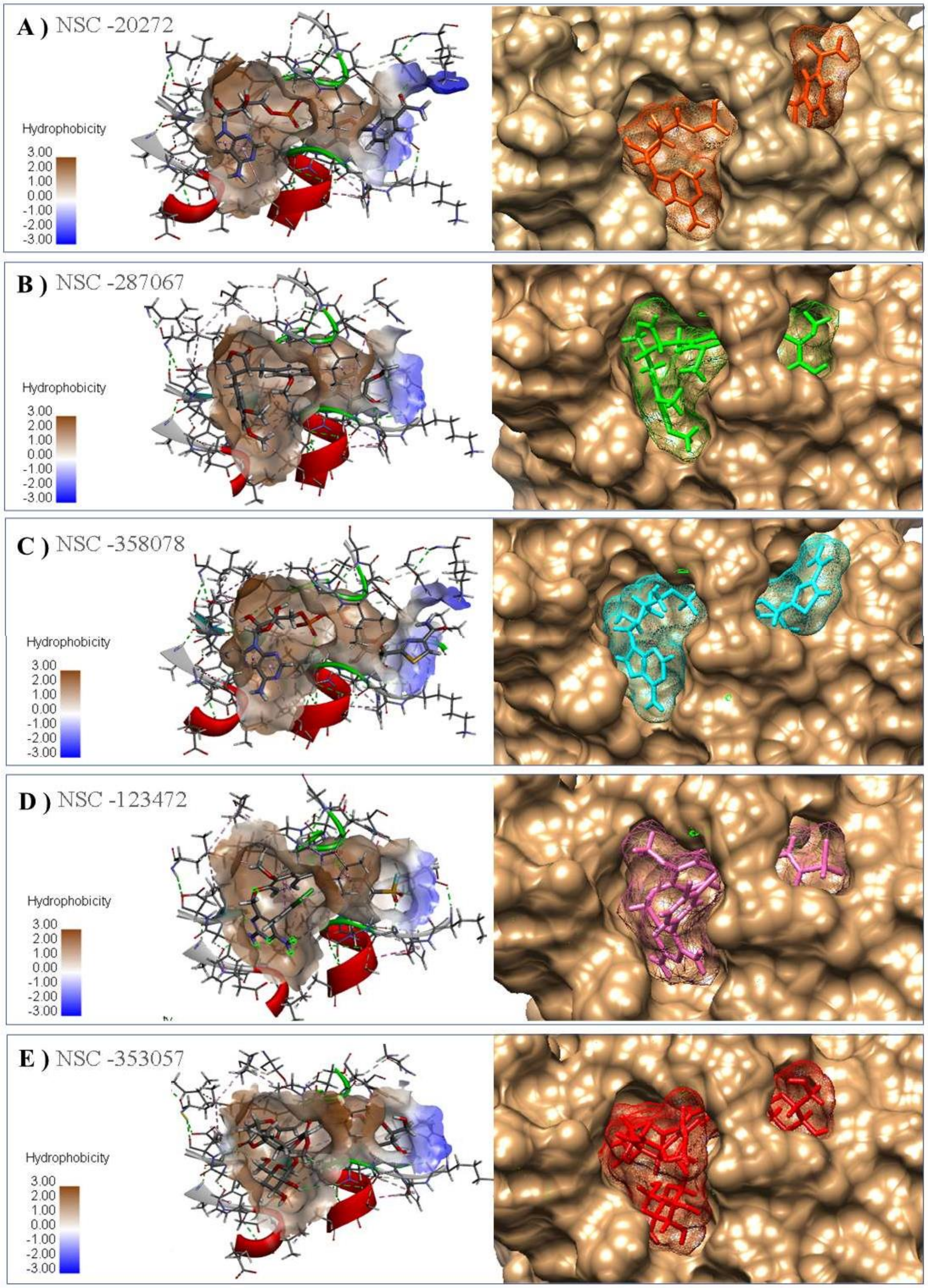

Figure S1. The 3D representation of the top scored NSC hits inside the cavity of Mac1 domain. The hydrophobic interactions have been demonstrated inside the cavity as well as the surface view of the docked complexes. The 3D docked conformations of the hits inside the ADP-ribose binding site in Mac1 domain have been also shown using Chimera and Discovery Studio software.

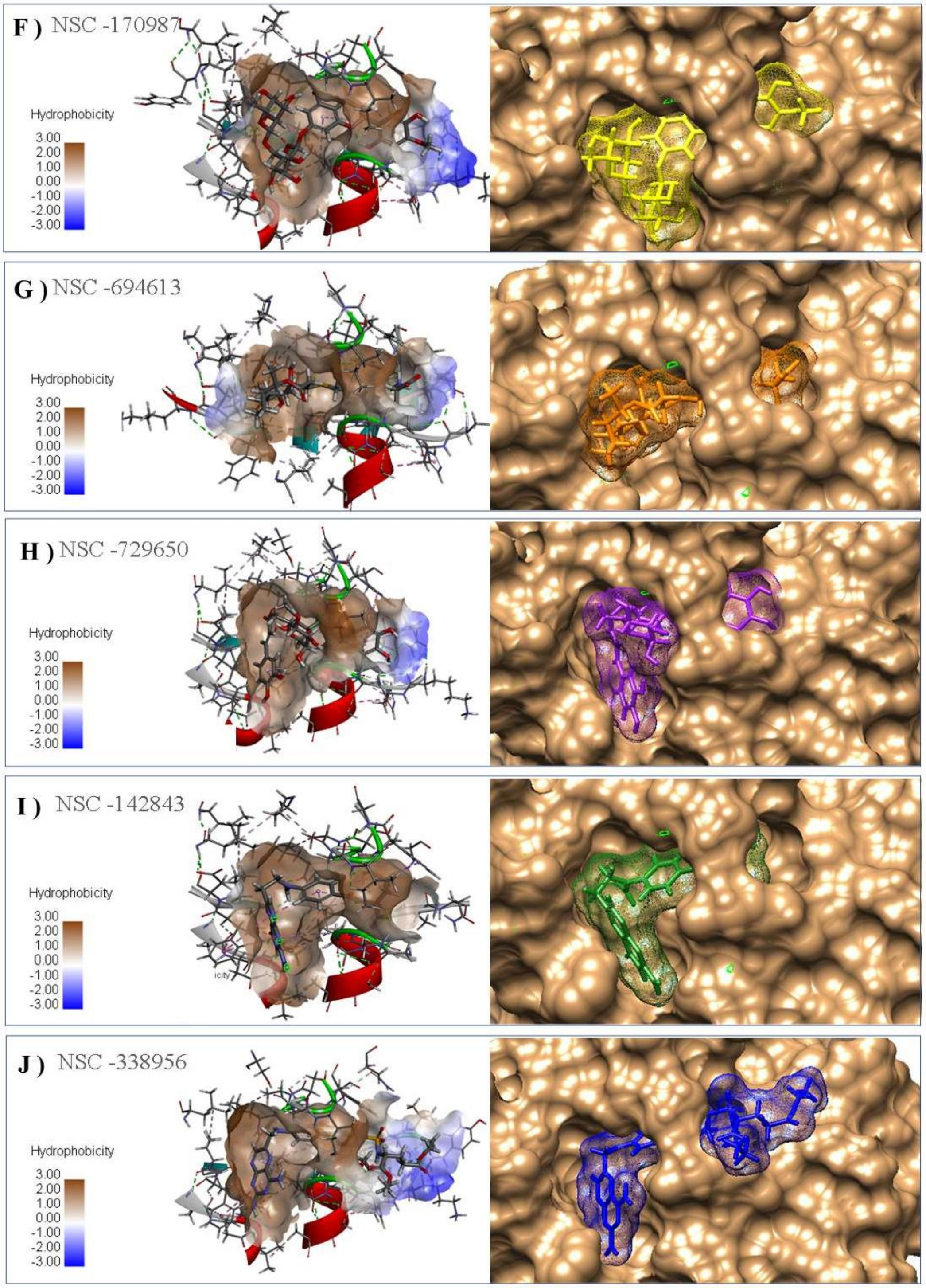

Figure S2. The 3D representation of the top scored NSC hits inside the cavity of Mac1 domain. The hydrophobic interacions have been demonstrated inside the cavity as well as the surface view of the docked complexes. The 3D docked conformations of the hits inside the ADP-ribose binding site in Mac1 domain have been also shown using Chimera and 37 Discovery Studio software.

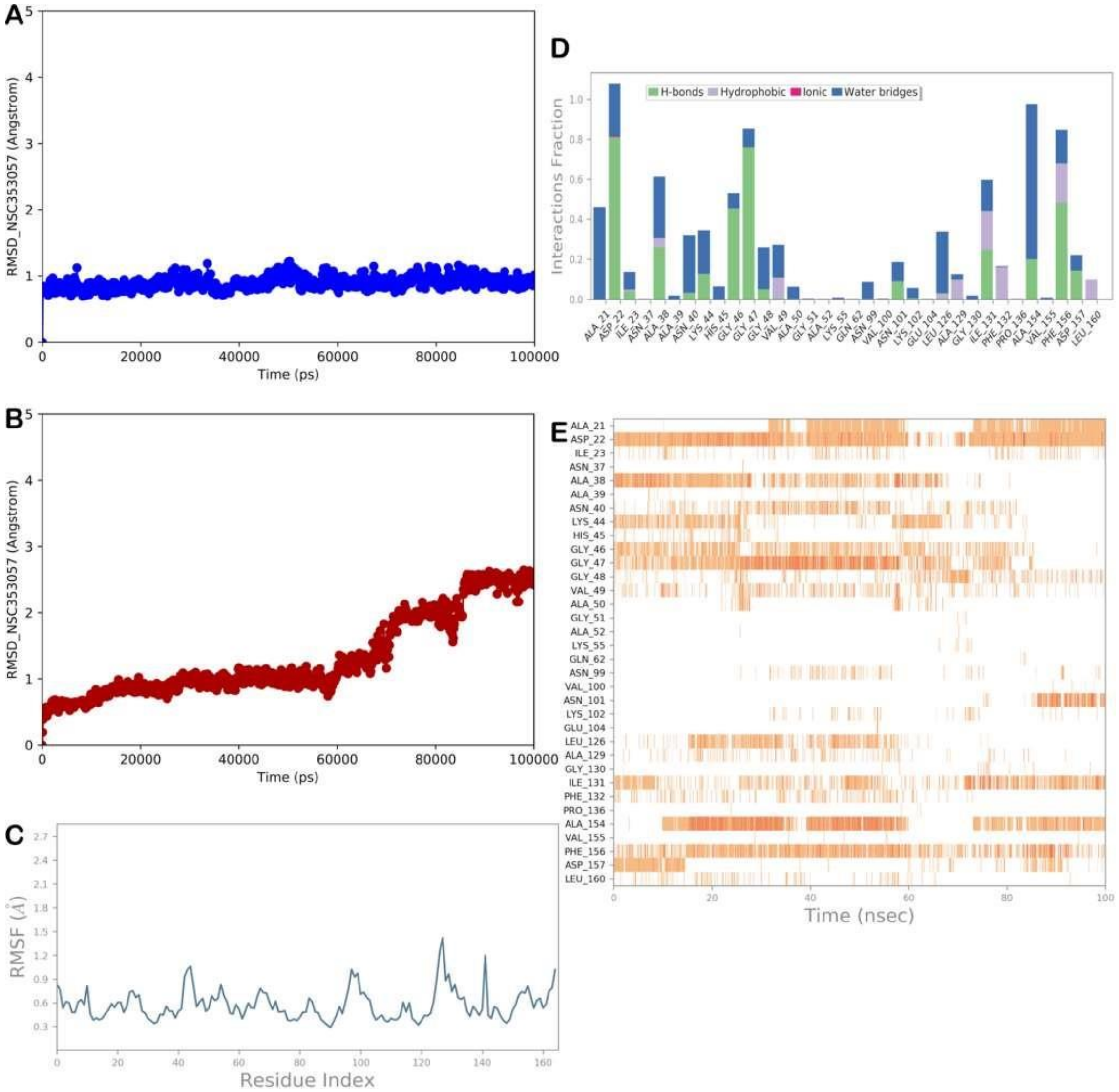

Figure S3. MD analysis of compound NSC-353057. (A,B) RMSD of the protein (blue line) and of the ligand (red line); (C) RMSF analysis of the protein; (D,E) NSC-353057 monitored during the MD run. The interactions can be grouped into four types: H bonds (green), hydrophobic (gray), ionic (magenta), and water bridges (blue). The stacked bar charts are normalized over the course of the trajectory: for example, a value of 0.7 suggests that, for 70% of the simulation time, the specific interaction is maintained. Values over 1.0 are possible, as some protein residue may make multiple contacts of the same subtype with the ligand. The subsequent diagram in the figure illustrates a timeline description of the main interactions. The output shows which residues interact with the ligand in each trajectory frame. Some residues make more than one specific contact with the ligand, which is represented by a darker shade of orange (Maestro, Schrödinger LLC, release 2020-3).

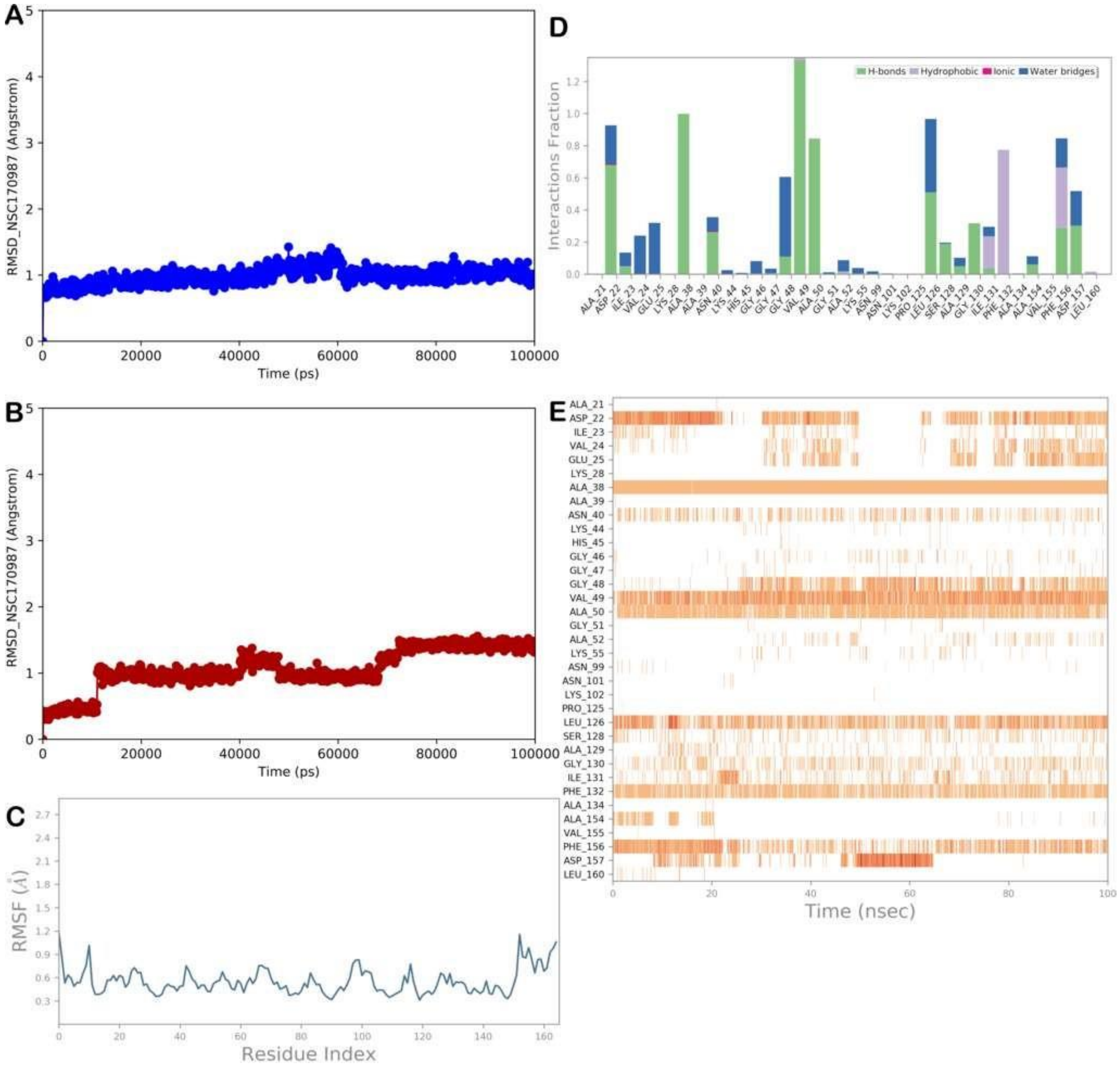

Figure S4. MD analysis of compound NSC-170987. (A,B) RMSD of the protein (blue line) and of the ligand (red line); (C) RMSF analysis of the protein; (D,E) NSC-170987 monitored during the MD run. The interactions can be grouped into four types: H bonds (green), hydrophobic (gray), ionic (magenta), and water bridges (blue). The stacked bar charts are normalized over the course of the trajectory: for example, a value of 0.7 suggests that, for 70% of the simulation time, the specific interaction is maintained. Values over 1.0 are possible, as some protein residue may make multiple contacts of the same subtype with the ligand. The subsequent diagram in the figure illustrates a timeline description of the main interactions. The output shows which residues interact with the ligand in each trajectory frame. Some residues make more than one 58 specific contact with the ligand, which is represented by a darker shade of orange (Maestro, Schrödinger LLC, release 59 2020-3).

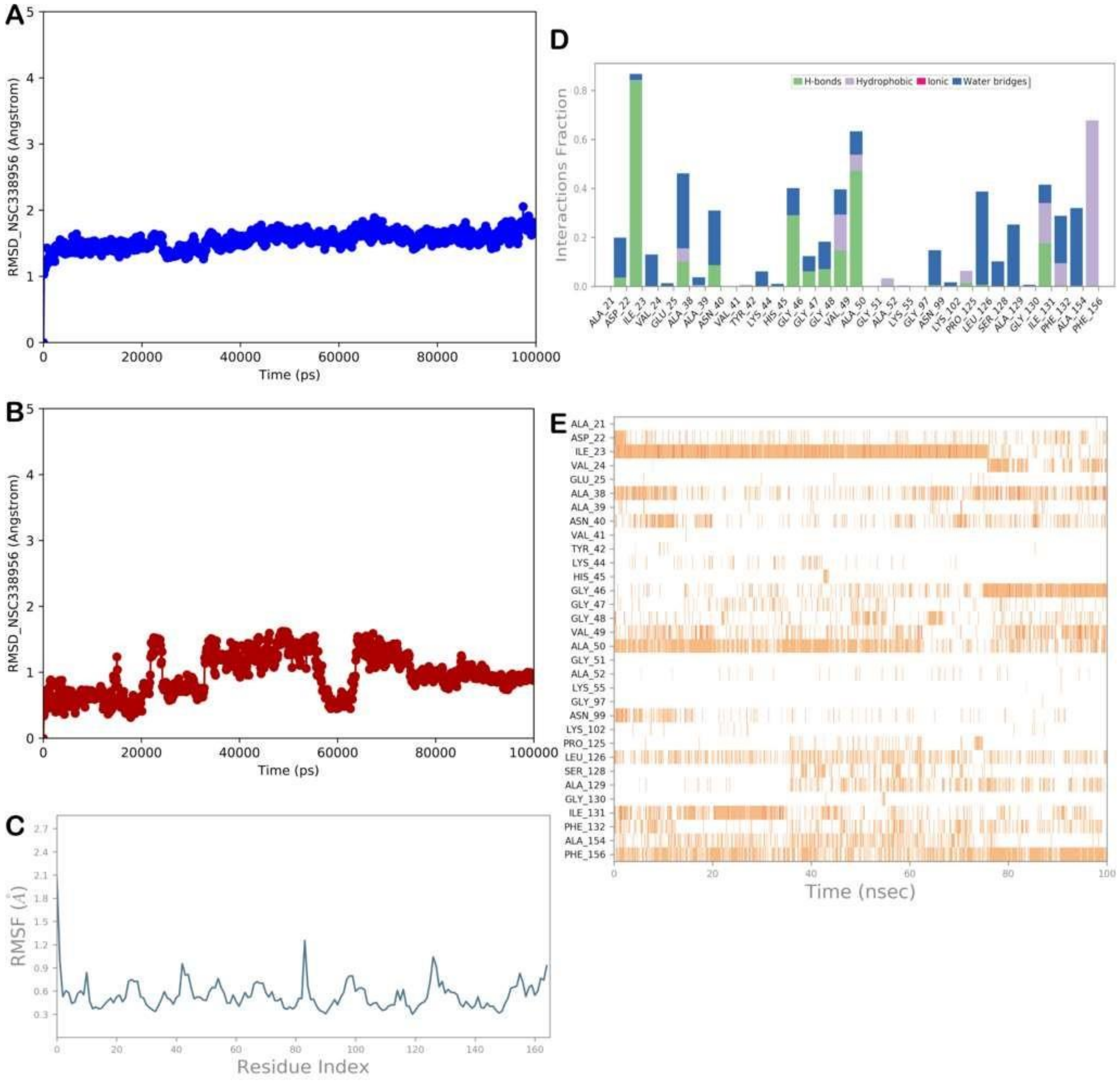

Figure S5. MD analysis of compound NSC-338956. (A,B) RMSD of the protein (blue line) and of the ligand (red line); (C) RMSF analysis of the protein; (D,E) NSC-338956 monitored during the MD run. The interactions can be grouped into four types: H bonds (green), hydrophobic (gray), ionic (magenta), and water bridges (blue). The stacked bar charts are normalized over the course of the trajectory: for example, a value of 0.7 suggests that, for 70% of the simulation time, the specific interaction is maintained. Values over 1.0 are possible, as some protein residue may make multiple contacts of the same subtype with the ligand. The subsequent diagram in the figure illustrates a timeline description of the main interactions. The output shows which residues interact with the ligand in each trajectory frame. Some residues make more than one specific contact with the ligand, which is represented by a darker shade of orange (Maestro, Schrödinger LLC, release 2020-3).

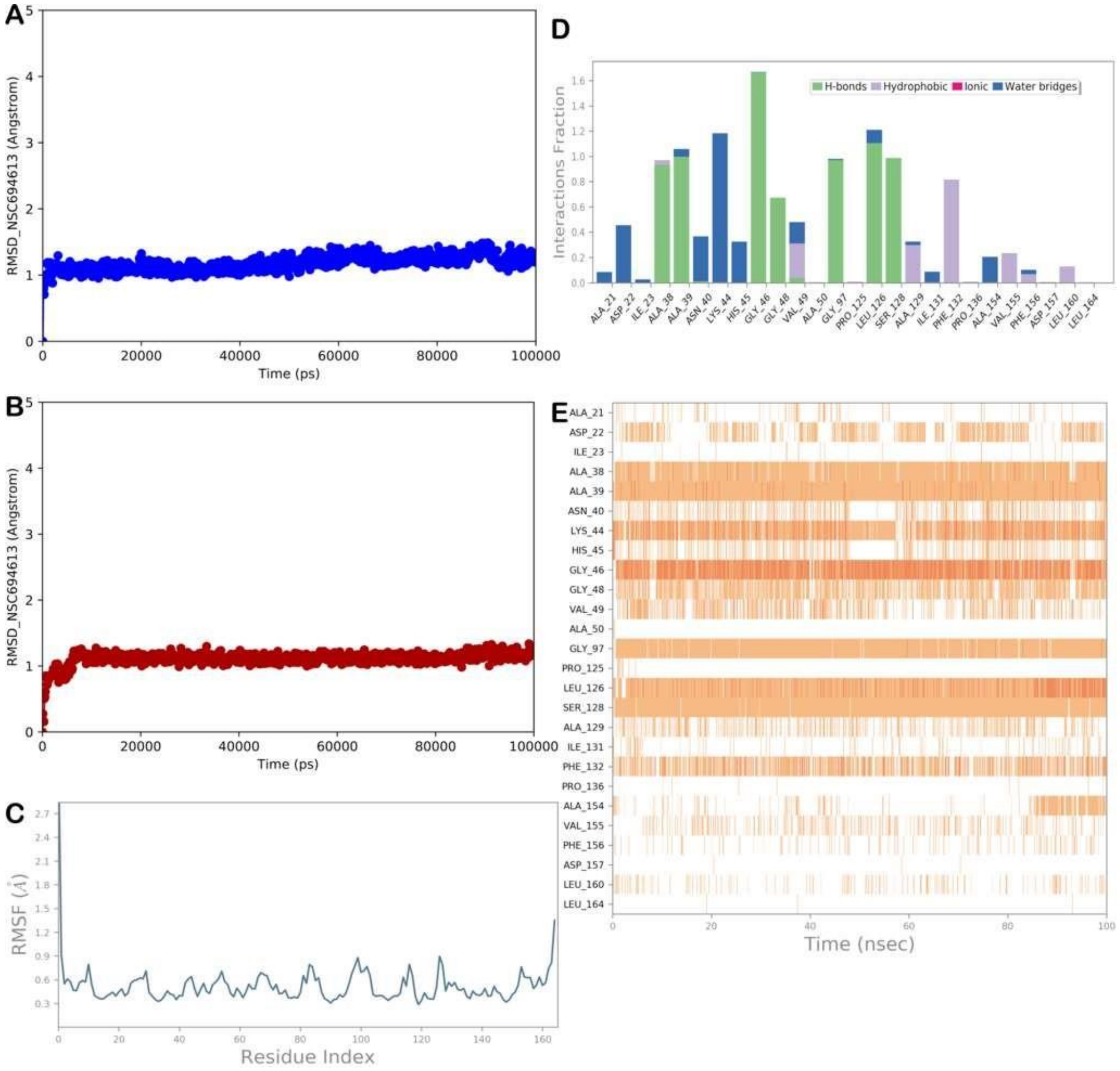

Figure S6. MD analysis of compound NSC-694613. (A,B) RMSD of the protein (blue line) and of the ligand (red line); (C) RMSF analysis of the protein; (D,E) NSC-694613 monitored during the MD run. The interactions can be grouped into four types: H bonds (green), hydrophobic (gray), ionic (magenta), and water bridges (blue). The stacked bar charts are normalized over the course of the trajectory: for example, a value of 0.7 suggests that, for 70% of the simulation time, the specific interaction is maintained. Values over 1.0 are possible, as some protein residue may make multiple contacts of the same subtype with the ligand. The subsequent diagram in the figure illustrates a timeline description of the main interactions. The output shows which residues interact with the ligand in each trajectory frame. Some residues make more than one specific contact with the ligand, which is represented by a darker shade of orange (Maestro, Schrödinger LLC, release 2020-3).

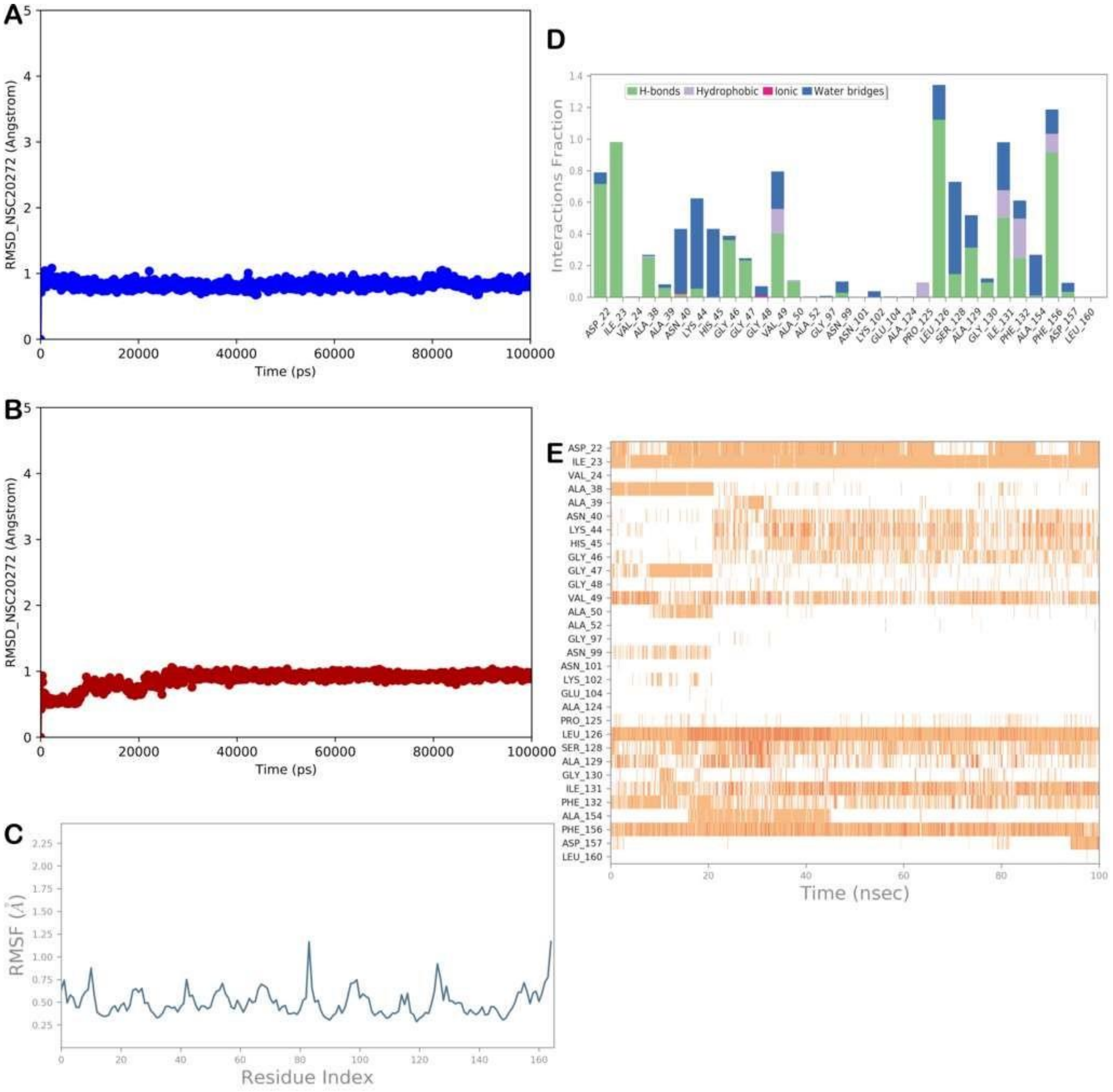

Figure S7. MD analysis of compound NSC-20272. (A,B) RMSD of the protein (blue line) and of the ligand (red line); (C) RMSF analysis of the protein; (D,E) NSC-20272 monitored during the MD run. The interactions can be grouped into four types: H bonds (green), hydrophobic (gray), ionic (magenta), and water bridges (blue). The stacked bar charts are normalized over the course of the trajectory: for example, a value of 0.7 suggests that, for 70% of the simulation time, the specific interaction is maintained. Values over 1.0 are possible, as some protein residue may make multiple contacts of the same subtype with the ligand. The subsequent diagram in the figure illustrates a timeline description of the main interactions. The output shows which residues interact with the ligand in each trajectory frame. Some residues make more than one specific contact with the ligand, which is represented by a darker shade of orange (Maestro, Schrödinger LLC, release 2020-3).

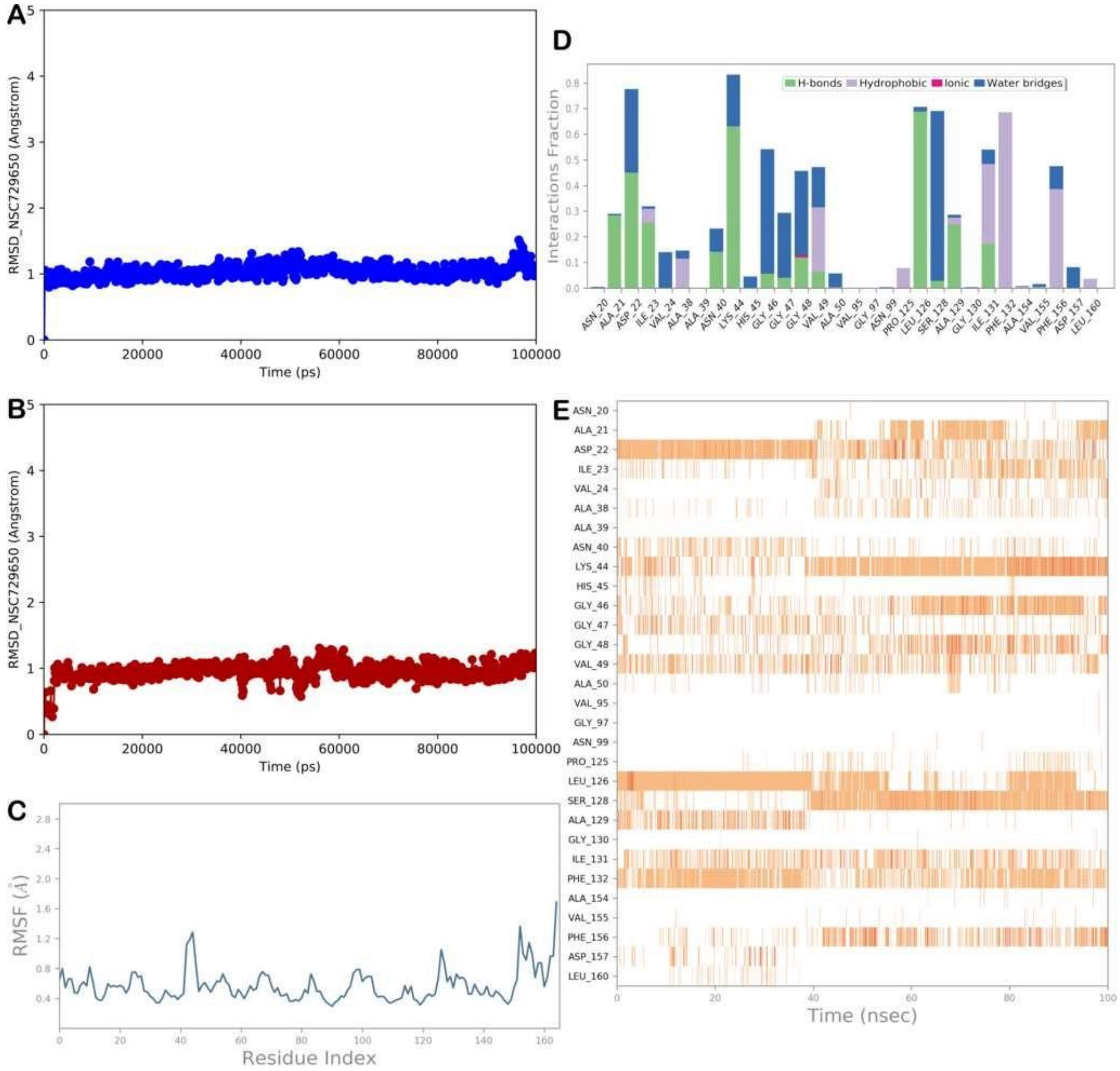

Figure S8. MD analysis of compound NSC-729650. (A,B) RMSD of the protein (blue line) and of the ligand (red line); (C) RMSF analysis of the protein; (D,E) NSC-729650 monitored during the MD run. The interactions can be grouped into four types: H bonds (green), hydrophobic (gray), ionic (magenta), and water bridges (blue). The stacked bar charts are normalized over the course of the trajectory: for example, a value of 0.7 suggests that, for 70% of the simulation time, the specific interaction is maintained. Values over 1.0 are possible, as some protein residue may make multiple contacts of the same subtype with the ligand. The subsequent diagram in the figure illustrates a timeline description of the main interactions. The output shows which residues interact with the ligand in each trajectory frame. Some residues make more than one specific contact with the ligand, which is represented by a darker shade of orange (Maestro, Schrödinger LLC, release 2020-3).
